# Structural Design and Sound Absorption Properties of Nitrile Butadiene Rubber-Polyurethane Foam Composites with Stratified Structure

**DOI:** 10.3390/polym10090946

**Published:** 2018-08-25

**Authors:** Xueliang Jiang, Zhijie Wang, Zhen Yang, Fuqing Zhang, Feng You, Chu Yao

**Affiliations:** 1 Hubei Key Laboratory of Plasma Chemistry and Advanced Materials, Wuhan Institute of Technology, Wuhan 430205, China; jiangxl@wit.edu.cn (X.J.); wzj0038@126.com (Z.W.); tabjpz@163.com (Z.Y.); zhangfq@wit.edu.cn (F.Z.); youfeng.mse@wit.edu.cn (F.Y.); 2 College of Materials Science and Engineering, Wuhan Institute of Technology, Wuhan 430205, China; 3 Ministry-of-Education Key Laboratory for the Green Preparation and Application of Functional Materials, Hubei University, Wuhan 430062, China

**Keywords:** NBR, PU foam, stratified structure, sandwich structure, sound absorption, low frequency

## Abstract

Sound absorbing composites with stratified structures, including double-layer and sandwich structures, were prepared through the combination of nitrile butadiene rubber (NBR) and polyurethane foam (PUFM). The effects of the thickness ratio of layers, different stratified structures and the variety of fillers on the sound absorption performance of the NBR-PUFM composites and the sound absorption mechanism were studied. The results show that the NBR-PUFM composite with a sandwich structure and thickness ratio of 1:8:1 displays good sound absorption, which could be improved further by adding fillers. Because the airflow resistivity, resonance absorption, interface dissipation and interface reflection were combined organically in the sandwich structure, the composites show excellent low-frequency sound absorption performance. Moreover, the composite also has advantages in cost and functionalization aspects.

## 1. Introduction

Currently, energy and the environment are receiving increased attention of researchers [1,2,3,4,5,6,7]. In modern industry, noise is one of the most urgent issues to be addressed, and the research of sound-absorbing materials is particularly critical. Structural design has always been an important method in the field of noise reduction. Different structures have different effects on acoustic absorption and sound transmission loss of composites. The preparation methods and the application of porous materials have attracted many researchers due to their special structure [8,9,10]. Maa [11] proposed a submicron micro-perforated panel sound absorbing structure that provided low acoustic mass and a sufficient amount of acoustic impedance to increase low-frequency absorption. Based on this, Duan [12] used flexible panel vibration effects to increase the sound absorption of micro-perforated panels at low frequency. Zhao [13] found that the parallel mechanical impedance structure could be utilized to increase the sound absorption at low frequency when this structure was arranged in cavity of a prefabricated micro perforated panel. The micro-perforated panel with the simple structure has a predictable absorption performance, but this composite is often affected by the acoustic waves caused by the material, and the acoustic absorption efficiency of the material is reduced due to the lightweight property of the micro-perforated panel [14]. In general, the soft porous foam materials with high open porosity are used in the field of noise reduction widely, due to their effective sound absorption property, low density, high acoustic resistivity and easy preparation of products with different shapes [15,16,17,18,19,20]. When sound propagates through theses pores, their energy can be absorbed through viscous and thermal losses. However, it is hard to control the pore size and the distribution of the produced cell structures because of the randomness of pore forming particles. The sound absorption efficiency of foaming materials is sensitive to characteristics of their porous structure, such as open porosity, cell size, microstructure of cell, and thickness of foams [21,22,23,24]. Therefore, the porous structure is strongly dependent on foaming technology and is difficult to regulate in practical applications. Moreover, merely depending on the porous structure means that sound absorption performance for low frequencies is difficult to increase effectively.

The combination of porous materials and resonant plates through structural design is also commonly used to improve the performance of sound absorption, in addition to optimizing the pore structure of foaming materials. Kim [25] proposed that multi-band sound absorption with a double resonant porous structure exhibited good low frequency sound absorption. Wang [26] presented a bionic coupling multilayer structure with impedance transfer and finite element methods, which significantly improved the low frequency absorption performance. Lee [27] found that the non-woven polyethylene terephthalate fabrics/polypropylene (PP) led to remarkable improvement of absorption coefficient compared with the plain PP board. Sun [28] reported that the foam/film sheet with an alternating soft/soft layers structure had better sound absorption characteristics than traditional single layer materials. In recent years, spiral structures [29], 3D-printed meta-structures [30] and other new structures have appeared, and relevant sound-absorbing composites with small thickness and low weight also have arisen. In our previous research, a new multilayered structure of sound-absorbing composites consisting of polyurethane foam and BaTiO_3_/nitrile butadiene rubber showed fine low-frequency sound absorption performance [31,32]. However, barium titanate, used as a filler, is expensive and the preparation of the multilayered structure is difficult, which impedes practical application. These structural composites not only increase the sound transmission loss due to the interface resonance, but also increase the interface reflection of sound waves, which contributes to sound absorption [33,34,35,36].

In this paper, nitrile butadiene rubber-polyurethane foam (NBR-PUFM) composites with stratified structures, including double-layer and sandwich structures, were prepared through structural design and formulation optimizing. The effects of the thickness ratio of layers, different stratified structures and the variety of fillers on the sound absorption performance of the NBR-PUFM composite were studied. Compared with the literature, the mechanism and the effect of sound absorption were analyzed, and cost control was also discussed.

## 2. Experimental

### 2.1. Materials

BaTiO_3_ powders (BT) with micrometer size were synthesized by the authors. Nitrile butadiene rubber (NBR), rare earth oxides of micrometer size, lead zirconate titanate (PZT) of micrometer size, carbon-white black (CW), polyurethane foam (PUFM) with density of 36 kg/m^3^, carbon black (CB), calcium carbonate (CaCO_3_), talc and other compounding ingredients were purchased locally.

### 2.2. Preparation of NBR-PUFM Composites

**Figure 1:**
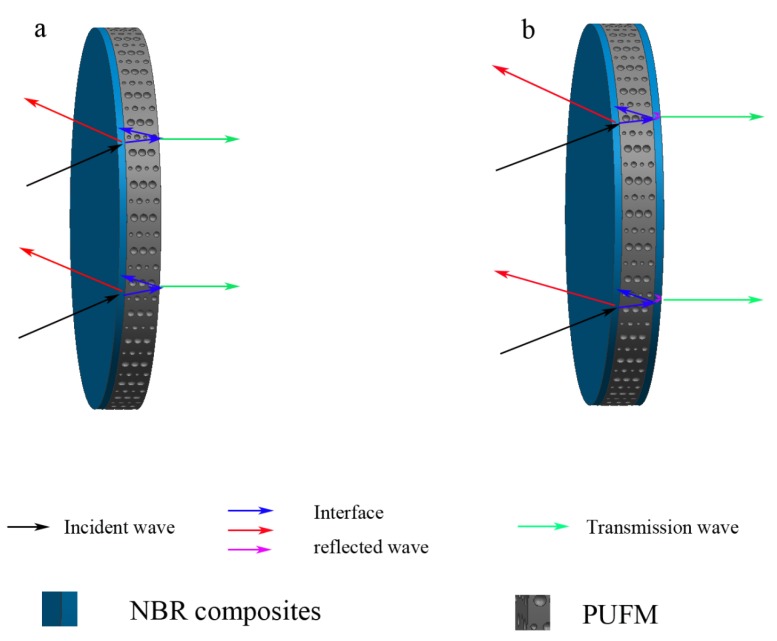
Schematic diagram of the NBR-PUFM composites with different stratified structures: (**a**) double-layer structure; (**b**) sandwich structure.

**Figure 2:**
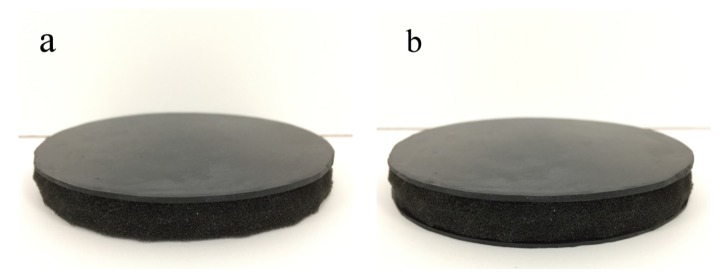
Actual shape of the NBR-PUFM composites: (**a**) double-layer structure; (**b**) sandwich structure.

NBR and the additives were initially mixed for 25 min in an open mill; formulations of the compounds are shown in Table 1. Additives were added in the order listed in Table 1. NBR composites were then placed at normal temperature for one day and vulcanized at 160 °C and 15 MPa for 15 min with a plate vulcanization machine (TZ112, Shanghai Tianzhi Shiye Co. Ltd., Shanghai, China). Surfaces of the NBR composites and the PUFM were then sprayed uniformly with a GUERQI V5 adhesive. After 30 s, the NBR composites were combined with the PUFM to prepare the double-layer structure composites and the sandwich structure composites (as in Figure 1). The thickness and diameter of the above two kinds of composites with different stratified structures was 10 and 100 mm, respectively. Figure 2 shows the actual shape of the samples with the double-layer and sandwich structures.

### 2.3. Characterization

Optical microscope (Nanjing Jiangnan Novel Optics Co. LTD, Nanjing, China) was utilized to obtain the morphology of the PUFM layers. The granularity analysis tool (Nano measurer) was used to analyze the visual image and determine the cell size distribution. The sound absorption coefficient (SAC) was tested using the standing wave tube method (AWA6128A, Beijing Century JT Technology Development Co. LTD, Beijing, China), which is the normal incidence sound absorption determination method. In the standing wave tube, a standing wave sound field would form after the incident and reflected wave on the surface are superposed. Through testing the ratio of maximum sound pressure to minimal sound pressure, the vertical incident sound absorption coefficients were calculated. The frequency determination was conducted at 11 frequency points in 200–2000 Hz according to 1/3-octave band.

## 3. Results

### 3.1. Morphology Analysis of the PUFM

**Figure 3:**
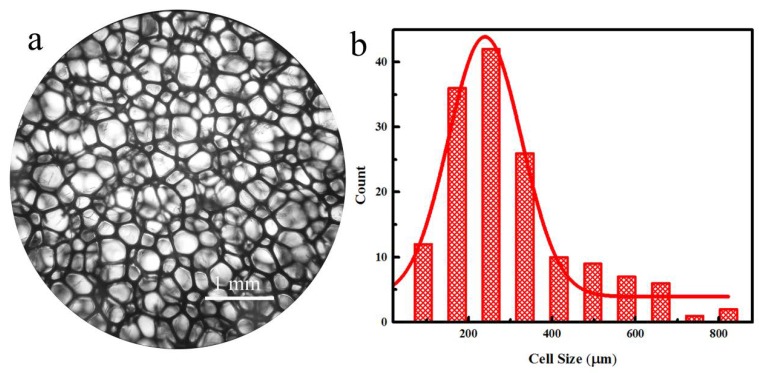
(**a**) Cell morphology and (**b**) cell size distributions of PUFM composites.

The cell structure of foam materials is directly relevant to their acoustic performance [15]; thus, the cell structure of PUFM is an important parameter of the NBR-PUFM sound-absorbing composites. The cell morphology of the PUFM was observed and the cell size distribution of the PUFM was obtained, as in Figure 3. Figure 3a shows that the porous structure of the PUFM contains a large amount of open pore structure and the diameters of the cells are relatively uniform. From Figure 3b, it can be seen that most of the cells have a diameter in the range of approximately 200 to 400 μm and the average cell diameter is 301 μm.

### 3.2. Sound Absorption Properties of the NBR-PUFM Composites

#### 3.2.1. Effect of the Thickness Ratio on SAC of the NBR-PUFM Composite with Double-Layer Structure

**Figure 4:**
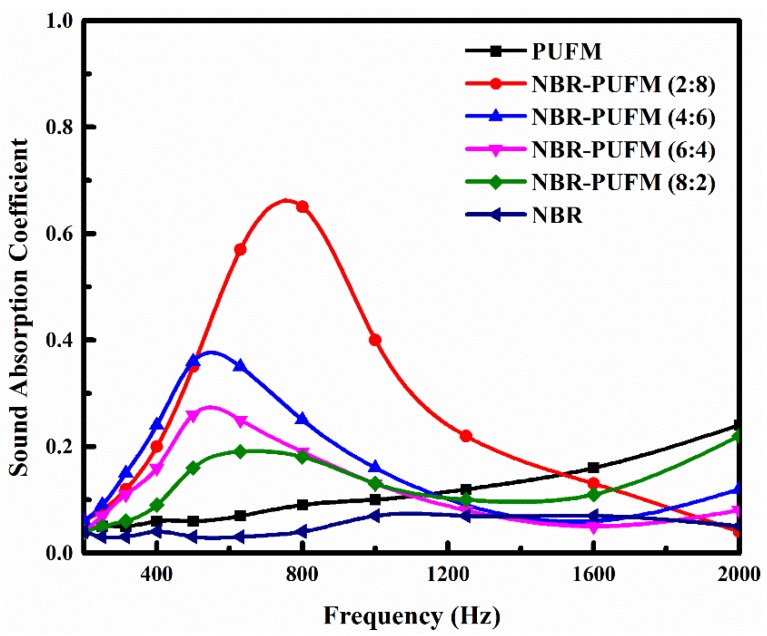
Effect of the thickness ratio on SAC of the NBR-PUFM composite with double-layer structure.

The thickness ratio of NBR and PUFM layers influences sound absorption; the influence of the thickness ratio on the SAC of the NBR-PUFM composite with double-layer structure is shown in Figure 4. It can be seen that both the NBR and the PUFM show low SAC in the frequency range, and their characteristic absorption peaks are not obvious. In contrast, the NBR-PUFM composites with double-layer structure exhibit fine sound absorption performance. With the increase of the thickness of the PUFM layer, the sound-absorbing composite shows better performance in a wider bandwidth of frequency, and the average SAC in the frequency range of 500 to 1600 Hz increases from 0.14 to 0.37 (as in Table 2). With the increase of the thickness of NBR layer, the characteristic peak of sound absorption moves to low frequency first, and then to high frequency. The composite shows best performance of sound absorption with the thickness ratio of 2:8, and its characteristic sound absorption peak shows lowest frequency with the thickness ratio of 4:6.

#### 3.2.2. Effect of the Thickness Ratio on SAC of the NBR-PUFM Composite with Sandwich Structure

**Figure 5:**
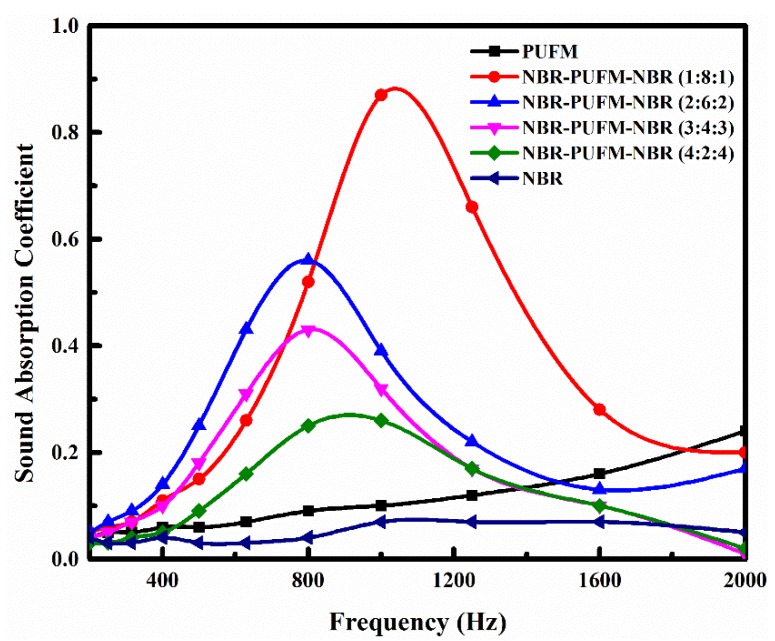
Effect of the thickness ratio on SAC of the NBR-PUFM composite with sandwich structure.

Figure 5 shows the effect of the thickness ratio of NBR layer, PUFM layer and NBR layer on the SAC of the NBR-PUFM composite with sandwich structure. With the increase of the thickness of PUFM layer, the composite shows better effect of sound absorption in a wider bandwidth of frequency, and the average SAC in the frequency range of 500 to 1600 Hz increases from 0.19 to 0.53 (as in Table 2). With the increase of the thickness of NBR layer, the characteristic peak of sound absorption moves to low frequency first, and then to high frequency. The composite shows best performance of sound absorption with the thickness ratio of 1:8:1, and its characteristic sound absorption peak shows lowest frequency with the thickness ratio of 2:6:2. The total thickness of NBR-PUFM composite is in the condition of invariability, whether the structure is double-layer or sandwich. However, the NBR-PUFM composite with sandwich structure obviously shows better performance of sound absorption than that with double-layer structure. It follows that it is an effective way to improve sound absorption efficiency at medium-to-low frequency range of materials through a simple sandwich structural design.

#### 3.2.3. Effect of the Fillers on SAC of the NBR-PUFM Composite with Sandwich Structure

**Figure 6:**
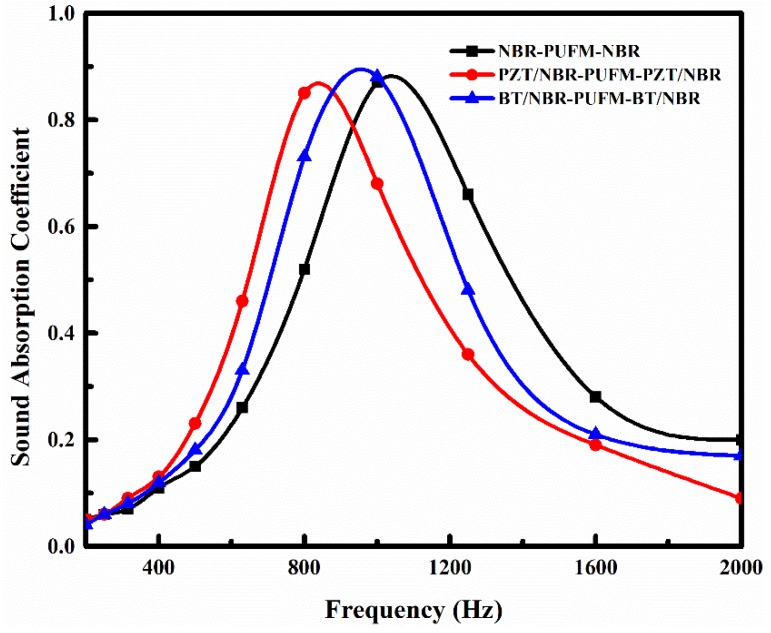
SAC of the sandwich structure NBR-PUFM composites after adding piezoelectric ceramics.

**Figure 7:**
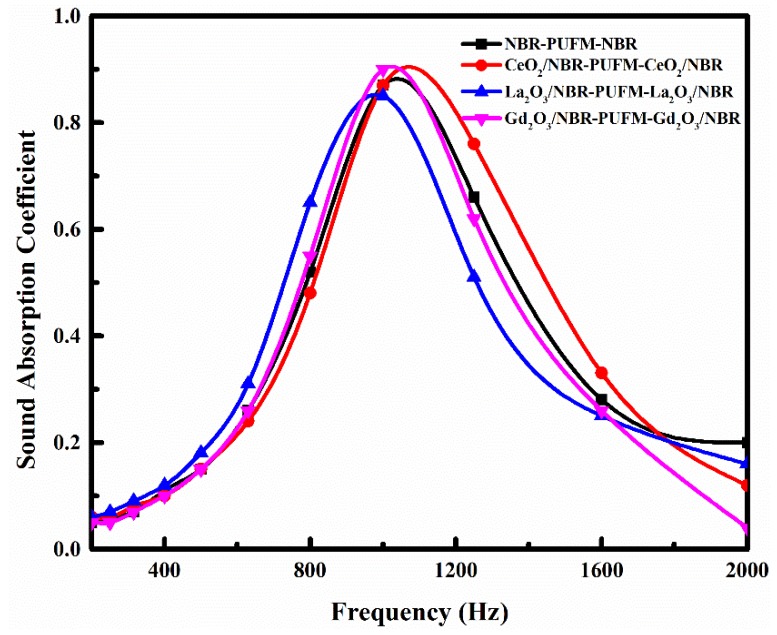
SAC of the sandwich structure NBR-PUFM composites after adding rare earth oxides.

**Figure 8:**
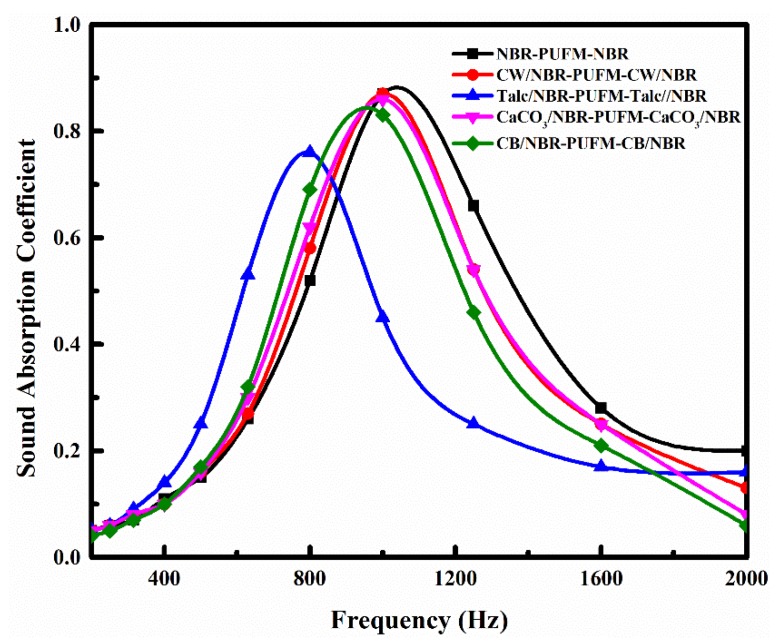
SAC of the sandwich structure NBR-PUFM composites after adding common fillers.

To obtain different NBR composites for the preparation of the NBR-PUFM composites with a sandwich structure and a thickness ratio of 1:8:1, various fillers were separately doped into the NBR; the effect of fillers on the sound absorption property of the NBR-PUFM composite with sandwich structure was then studied. Figure 6, Figure 7 and Figure 8 show the sound absorption performance of the composites filled with piezoelectric ceramics, rare earth oxides and common fillers, respectively. The results indicate that the characteristic absorption peak of sound waves moves to low frequency after adding the vast majority of the fillers, including some piezoelectric ceramics (such as PZT and BT), some rare earth oxides (such as La_2_O_3_ and Gd_2_O_3_), and some common fillers (such as CW, talc, CaCO_3_ and CB). The sound absorption performance in low frequency can be improved by adding fillers. However, the average absorption coefficients of the NBR-PUFM composite with sandwich structure in the frequency range of 500 to 1600 Hz showed little change after adding the fillers (as in Table 3). It is worth noting that the use of low-cost fillers such as CW, talc, CaCO_3_ and CB not only improves the low frequency sound absorption performance of composites, but also greatly reduces the preparation cost of composites and broadens the development prospects.

## 4. Discussion

In order to discuss the sound-absorption mechanisms of the stratified structure NBR-PUFM composites, the roles of NBR layer and PUFM layer should first be confirmed in the absorption process of sound waves. With the increase of the thickness of NBR layer, the sound absorption performances of the NBR-PUFM composites with both double-layer and sandwich structures decreased (as in Figure 4 and Figure 5). Therefore, the PUFM layer played a major role in the process of sound absorption. However, PUFM could not effectively absorb the low frequency sound waves alone. This is because low frequency sound wave has strong penetrability and is hard to absorb compared with medium-high frequency sound waves. PUFM would give priority to medium-high frequency sound waves in the process of sound absorption, so the low frequency sound wave was able to penetrate the PUFM. However, sound absorption by PUFM was different after NBR was combined with PUFM. The NBR layer, a layer with relatively high density, reflected a significant quantity of the sound waves in the interface of air and the NBR layer, especially medium and high frequency sound waves [31,32]. Therefore, most of the low frequency sound waves, and a few medium and high frequency sound waves, passed through the NBR layer and entered the interior of the PUFM layer. PUFM would absorb the low frequency sound waves effectively in this situation. In summary, the NBR layer played a role of improving the airflow resistivity to isolate the medium-high frequency sound waves, and the PUFM layer played the role of absorbing the low frequency sound waves.

For the sandwich structure, there are several mechanisms we note that can explain the improvement of sound absorption performance. On the one hand, the NBR-PUFM composites absorbed sound energy through the resonance of the mass-spring system, which was composed of the NBR layer as the mass and the PUFM layer as the spring. The sound waves entered the NBR-PUFM composites and caused the resonance of the combined stiffness of NBR with the compressible skeleton frame of PUFM and the air in the pores of PUFM, leading to various energy losses in the system. On the other hand, the interface between adjacent layers with different acoustic impedance had a beneficial effect on the sound absorption [33,34]. Because of the impedance mismatch, the probability that the sound waves were reflected by the interface increased. Therefore, the sound propagation path in the sound absorption composites increased in length, which led to more air friction and viscoelastic damping dissipation in the PUFM layer. Compared with the double-layer structure, the sandwich structure has an additional interface of NBR and PUFM layers. Hence, more interface resonances occurred, and some sound waves which passed through the PUFM layer were reflected and reentered the interior of the PUFM layer. Due to the aforementioned reasons, the sandwich structure could enhance the sound absorption performance. Furthermore, compared with the double-layer structure where the bubble holes are exposed, the sandwich structure has distinctive superiority in both flame retardation [37], waterproofing [38] and shock resistance [39], since both outside layers were designed as solid rather than porous structures.

Adding the filler into the NBR layer could improve the low-frequency SAC of the NBR-PUFM composite, but the variety of fillers has a weak impact on sound absorption property (as in Figure 6, Figure 7 and Figure 8). Thus, it can be concluded that the relatively higher density being provided by the fillers is the main reason for the improvement of sound absorption, rather than functional aspects of fillers, such as piezoelectric and magnetic effects. The density of the NBR layer improved after the filler was added, leading to more reflection of sound waves in the interfaces of the NBR and PUFM layers. More reflection of sound waves would result in a longer sound propagation path. Therefore, the NBR-PUFM composite dissipated the energy of sound waves more effectively through resonance absorption of filler particles and more interface reflection of the sound waves. In addition, when NBR was combined with fillers, the varying acoustic mass might make hybrid resonances impedance-matched with airborne sound at tunable frequencies, which was another reason causing the improvement of low frequency sound absorption performance [40]. In summary, the filler benefits sound absorption of the NBR-PUFM composite, as long as the filler could increase the density of the composite. Therefore, low-cost fillers, such as CW, talc, CaCO_3_ and CB, could reduce the cost of the NBR-PUFM composite effectively compared with expensive piezoelectric ceramics and rare earth oxides. Moreover, it is convenient to prepare the NBR-PUFM composite with good low frequency sound absorption performance and special functionality via various fillers, such as flame-retardant filler, extending filler, heat-insulating fillers, and so on.

In previous studies, the simple combinations of polymers and fillers such as mica powder [41], graphene foam [42], and mesoporous silica [43] could not significantly increase the sound absorption of materials at low frequency. In particular, the SAC was hard to increase at a frequency below 1000 Hz. Moreover, while the alternating soft/soft layers structure [28], the spiral structure [29], the 3D-printed meta-structure [30] and the porous structure [15] have been reported as increasing the SAC and broadening the absorption frequency band, the effect of sound absorption was not found to be sufficient at low frequency. The NBR-PUFM composite studied in this paper shows excellent sound absorption at low frequency. As discussed, this sound absorption composite could have additional characteristics—such as lower cost, flame retardation, heat preservation, thermal insulation, water-tightness, and so on—depending on its sandwich structure and addition of various fillers. Moreover, it was found that the sound absorption performances of the NBR-PUFM composites with both double-layer and sandwich structures increased with the decrease of the thickness ratio of the NBR layer and the PUFM layer; densities of the NBR layer and the PUMF layer are 1.099 × 10^3^ and 0.036 × 10^3^ kg/m^3^, respectively. Therefore, the NBR-PUFM composites could have both excellent sound absorption performance and low weight. In summary, it could be expected that NBR-PUFM composites with a sandwich structure could have broad development prospects in the automobile industry, building materials industry and petrochemical industry.

## 5. Conclusions

NBR-PUFM composites with stratified structures, including double-layer and sandwich structures, were prepared by the combination of NBR and PUFM. With the increase of the thickness of the NBR layer, the sound absorption performance of the NBR-PUFM composites with both double-layer and sandwich structures decreased. The NBR-PUFM composite with a sandwich structure and thickness ratio of 1:8:1 displayed good sound absorption, which could be improved further by adding fillers that provided relatively higher density and airflow resistivity. The NBR layer played the role of improving airflow resistivity to isolate medium-high frequency sound waves, and the PUFM layer played the role of absorbing low-frequency sound waves. Due to the sandwich structure there was greater air friction, viscoelastic damping dissipation, interface resonance, and interface reflection, which could dissipate the energy of sound waves effectively. Moreover, the NBR-PUFM composite with a sandwich structure also has advantages in cost and functionalization aspects.

## Figures and Tables

**Table 1:** Formulation of the compounds.

Content (phr)	Piezoelectric Ceramics		Rare Earth Oxides			Common Fillers			
	BT	PZT	CeO_2_	La_2_O_3_	Gd_2_O_3_	CW	Talc	CaCO_3_	CB
NBR	100		100			100			
ZnO	5		5			5			
Stearic acid	1.5		1.5			1.5			
Tetramethyl thiuram disulphide	2		2			2			
*N*-Cyclohexyl-2-benzothiazole sulfenamide	1		1			1			
*N*-isopropyl-*N*′-phenyl-*p*-phenylene	1		1			1			
Conductive carbon black	2		0			0			
CB	18		20			20	20	20	45
BT	25	0	0			0			
PZT	0	25	0			0			
CeO_23_	0		25	0	0	0			
La_2_O_3_	0		0	25	0	0			
Gd_2_O_3_	0		0	0	25	0			
CW	0		0			25	0	0	0
Talc	0		0			0	25	0	0
CaCO_3_	0		0			0	0	25	0
Sulfur	1.5		1.5			1.5			

**Table 2:** Maximum and average SAC of the NBR-PUFM composites in the frequency range of 500 to 1600 Hz.

Sound Absorption Coefficient (α)	NBR	PUFM	Double-Layer Structure (NBR:PUFM)				Sandwich Structure (NBR:PUFM:NBR)			
			2:8	4:6	6:4	8:2	1:8:1	2:6:2	3:4:3	4:2:4
**Maximum**	0.07	0.24	0.65	0.36	0.26	0.19	0.87	0.56	0.43	0.26
**Average**	0.06	0.11	0.37	0.18	0.14	0.14	0.53	0.32	0.25	0.19

**Table 3:** Maximum and average SAC of the sandwich structure NBR-PUFM composites after adding fillers in the frequency range of 500 to 1600 Hz.

Sound Absorption Coefficient (α)	Without Fillers	Piezoelectric Ceramics		Rare Earth Oxides			Common Fillers			
		PZT	BT	CeO_2_	La_2_O_3_	Gd_2_O_3_	CW	Talc	CaCO_3_	CB
Maximum	0.87	0.85	0.88	0.87	0.85	0.90	0.87	0.76	0.86	0.83
Average	0.53	0.49	0.52	0.56	0.52	0.53	0.51	0.40	0.52	0.50
